# The understudied links of the retromer complex to age-related pathways

**DOI:** 10.1007/s11357-021-00430-1

**Published:** 2021-08-09

**Authors:** Kenneth A. Wilson

**Affiliations:** Buck Institute for Research On Aging, 8001 Redwood Boulevard, Novato, CA 94947 USA

**Keywords:** Retromer complex, Aging, Protein trafficking, Neurodegeneration, Alzheimer’s disease, Parkinson’s disease, Autophagy

## Abstract

Neuronal aging is associated with numerous diseases resulting in memory impairment and functional decline. A common hallmark of these disorders is the accumulation of intracellular and extracellular protein aggregates. The retromer complex plays a central role in sorting proteins by marking them for reuse rather than degradation. Retromer dysfunction has been shown to induce protein aggregates and neurodegeneration, suggesting that it may be important for age-related neuronal decline and disease progression. Despite this, little is known about how aging influences retromer stability and the proteins with which it interacts. Detailed insights into age-dependent changes in retromer structure and function could provide valuable information towards treating and preventing many age-related neurodegenerative disorders. Here, we visit age-related pathways which interact with retromer function that ought to be further explored to determine its role in age-related neurodegeneration.

## Introduction

Neurodegenerative diseases such as Alzheimer’s, Parkinson’s, and Huntington’s are among the most prevalent age-related disorders [1–4]. These lead to loss of memory and motor control, and the associated decline in health is emotionally and financially costly [5]. Despite many hallmarks found for each of these diseases, such as the presence of amyloid-β extracellular plaques and intracellular hyper-phosphorylated Tau neurofibrillary tangles in Alzheimer’s disease or α-synuclein in Parkinson’s disease, there is no known way to prevent or cure many of them [6]. Clinical trials for treatments based on the clearance of these aggregates have proven disappointing [7], sparking investigations to find other means to stave off these age-related detriments.

Proper protein trafficking is essential for neuronal health as well as the processing of proteins which aggregate in neuronal decline [8]. In particular, endocytosed proteins must be sorted for degradation or reuse, and the balance between these two fates is intricate [9]. Proteins which are signaled for degradation are trafficked from the late endosome to the lysosome for autophagy. They can also be trafficked directly to the plasma membrane or the trans-Golgi network when sorted through the retromer complex [10], named for its function of facilitating retrograde transport in neurons [11]. Dysfunction in the retromer complex has been implicated in numerous defects, including developmental problems in simple organisms such as *Drosophila melanogaster* [12] and neurodegenerative phenotypes [13, 14]. It is also known to be important for age-related neuronal homeostasis [15], yet nothing is known about how the complex and its function are affected by age. How this mechanism declines, which mechanisms regulate its age-related function, and what it traffics to prevent neuronal decline are all questions that ought to be of paramount importance for understanding the mechanisms of neurodegeneration.

## The retromer mechanism

**Fig. 1:**
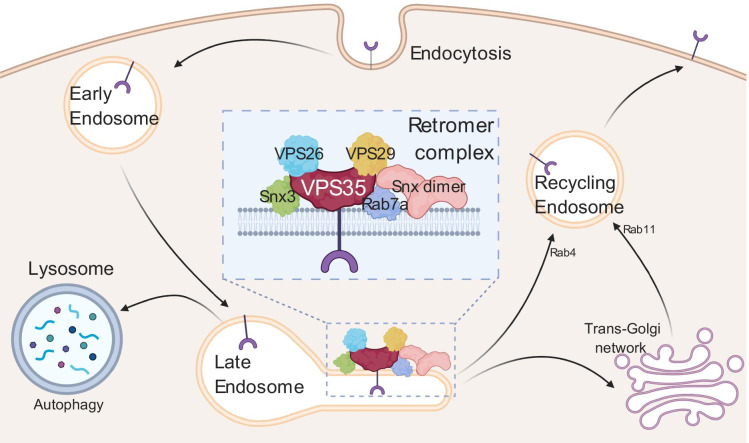
Endocytosis and the retromer complex. Proteins which are endocytosed are either degraded at the lysosome or are sorted by the retromer complex at the late endosome. Proteins sorted by the retromer are directed for fast transport or processing by the trans-Golgi network

The retromer complex localizes at the late endosome, interacting with the late endosomal protein Rab7 (Fig. 1) and other Rab7-interacting proteins such as TBC1D5 [16]. The complex core consists of a heterotrimer of vacuolar protein sorting 35 (VPS35), VPS26, and VPS29 [17]. This complex is responsible for recognizing cargo to be trafficked. Deletion of VPS26, VPS35, and in some contexts VPS29 can eliminate retromer-mediated trafficking altogether [12, 15, 18]. This trimer is conserved across species, and dysfunction of these components have been implicated in lifespan shortening and severe neurological defects in complex organisms [12, 19, 20].

A number of proteins interact directly with this trimer. Most prominently, sorting nexins are responsible for facilitating the trafficking of proteins. There are over 30 sorting nexins (SNXs) which interact with different stages of endocytosis and protein sorting [21]. Specifically, SNX1/2 [22] and SNX5/6 [23] as well as SNX3 [24] directly interact with the retromer to facilitate its activity. Depending on the specific SNXs which bind to the retromer, the cargo is then trafficked to different cellular loci [25, 26]. Proteins responsible for this trafficking include SNX27 [27], Rab4, which binds vesicles and cargo destined for the plasma membrane [27, 28], and Rab11, which mediates the trafficking of vesicles and cargo post-Golgi processing [29, 30].

## The retromer and neurodegenerative disease

The retromer complex has primarily been studied with reference to neurodegenerative diseases. Related to Alzheimer’s disease (AD), β-secretase (BACE) [31–34] and its cleavage target amyloid precursor protein (APP) [32, 35–37] are shuttled through the retromer complex. The cleavage of APP by BACE contributes to the toxic aggregation of amyloid-β. Stabilization of the retromer complex by the chaperone compounds R33 and R55 reduces the presence of amyloid-β [38, 39] as well as Tau neurofibrillary tangles [39]. VPS35 (D620N) mutations are associated with Parkinson’s disease (PD) phenotypes, dopaminergic neuron loss, and hippocampal Tau accumulation [40]. This association has been attributed to an interaction between VPS35 and LRRK2, a key protein which interacts with Parkin [41]. RNAi for VPS26 has also been shown to induce α-synuclein accumulation in fly neurons. Similar to AD phenotypes, this can be prevented with R55 treatment [12]. The retromer has also been implicated in Huntington’s disease (HD), as SorCS2, which is downregulated in R6/2 and zQ175 HD mouse models, which in turn downregulates VPS35 [42].

In addition to proteins which aggregate in specific diseases, the improper retromer-mediated sorting of other proteins has also been found to induce age-related neuronal decline. One of the earliest examples of retromer-mediated sorting was the trafficking of Wnt receptor. Wnt-mediated axon development was found to be severely impaired in nematode worms and frogs with retromer dysfunction [43, 44]. Improper Wnt function has been linked to AD [45] and PD [46], indicating the importance of properly regulating the components in Wnt signaling. Wnt signals induce the mitochondrial unfolded protein response and this process relies on retromer function [47], suggesting an essential role for the retromer in cellular stress response. Retromer function has also been linked to the trafficking of arrestins [48], which regulate signal transduction at GPCRs. Different types of arrestins regulate different cellular signals, including visual systems through photoreceptor signaling. Arrestin inhibition has been shown to alter photoreceptor morphology and light sensitivity [49] and arrestin function is also known to be altered by age [50]. The retromer complex has been specifically shown to be a key player in mediating beta-arrestin 1 function [48]. The relationship between the retromer’s regulation of arrestins with age remains unexplored, but could provide new insights into visual degeneration.

## Links to age-related pathways

Despite the clear importance of retromer function in age-related neurodegenerative diseases, little is known about how age influences the retromer. It has been shown that retromer protein levels are reduced in the cortex of 14-month-old transgenic AD mice (Tg2576) [51], but it remains unknown how it is affected by normal aging. There are, however, numerous age-related pathways which have been linked to retromer function. These pathways need to be further characterized to determine how they impact (or are impacted by) retromer activity.

One aspect of age-related retromer function that requires further study is the relationship between the retromer and age-related pathways. One such crossover has been observed between proteins of the Sortilin family. As previously mentioned, SorCS2 has been linked to retromer sorting and HD [42]. Another protein within the same protein family, SORL1, has also been extensively linked to retromer dysfunction-related amyloid-β aggregation and AD phenotypes [52, 53]. SORL1 has previously been implicated in diabetes, providing an interesting direction for future studies comparing the incidence of diabetes and AD [54]. Relating to this, SNX6 has also been found to physically interact with insulin-like growth factor 1 receptor (IGF1R) [55]. This also stimulates the phosphorylation of AKT, influencing growth and aging signals [55]. Additionally, the phosphoinositide binding domain (PX) in SNXs is recruited by phosphoatidylinositol-3-phosphate (PtdIns3P, or PI3P) at the endosomal membrane [56, 57]. PI3P activity is known to mediate signals downstream of AKT signaling upstream of retromer function [58]. This creates a challenge, as retromer function is necessary for healthy aging yet is regulated by age-promoting mechanisms. Interestingly, previous work has also demonstrated that insulin can induce retromer dissociation, which results in GLUT4 deficiency due to its over-degradation by the lysosome [59]. Taken together, these findings suggest that while retromer function can contribute to cell growth through insulin signaling, over-active insulin signaling can also cause retromer dysfunction. Significantly more dissection of this paradox is necessary to determine how to efficiently maintain retromer function without promoting mechanisms that contribute to aging.

Proper maintenance of the retromer complex also maintains healthy lysosomal function [18, 60, 61]. Studies using *Drosophila* models of retromer dysfunction have shown an abundance of aberrant lysosomes and photoreceptor necrosis [12, 61]. With fewer possible fates for endocytosed proteins in retromer dysfunction, it is possible that more proteins are being shuttled for autophagy, thus overburdening the lysosomes. This needs to be more extensively tested, but it provides an interesting counterpoint to many anti-aging therapies that aim for pathways which promote autophagy alone, such as dietary restriction and mTOR-targeting therapeutics like rapamycin. Interestingly, the only current link between mTOR activity and the retromer demonstrates that retromer activity is essential for mTOR to respond to dietary amino acids [62]. This suggests that retromer function contributes to mTOR activity, known to advance aging. These results indicate that retromer function surprisingly contributes to aging rather than providing an important function for age-related neuronal homeostasis [62]. Previously, it had also been shown that interaction between VPS35 and the Golgi-related protein GOLPH3 regulate mTOR activity [63], and that deletion of VPS29 and VPS35 influences sensitivity of cells to rapamycin [64]. There is a clear relationship between mTOR and retromer-related proteins, though the influence of this relationship on aging and disease progression remains unexplored. Additionally, though autophagy and retromer function are known to be dependent on each other for cell maintenance, it is not clear how both can be regulated in conjunction to maximize neuronal survival and health nor how mTOR can be related to this relationship.

## Regulators of retromer function

Retromer dysfunction has numerous outcomes due to it essential function, but it is worth noting that the most common resulting phenotypes are akin to age-related neurodegenerative diseases such as AD and PD. Despite clear links between age-related disease and the retromer complex, few factors are known which regulate retromer activity. One such factor that has been elucidated is phospholipase A2 group VI (PLA2G6), which is also linked to PD [12]. This study showed that VPS26 dysfunction led to accelerated photoreceptor decline with age in flies, and that this response was related to improper clearance of ceramides. They also showed direct interactions between PLA2G6, VPS26, and VPS35. PLA2G6 knockout caused α-synuclein accumulation which was alleviated by retromer stabilization. Similarly, CLN3 regulates retromer function through its interaction with Rab7 [65]. With such a clear relationship between aging, the retromer, and neuronal health, there is ample room for study to determine how retromer decline takes place and what factors regulate its function. There are likely numerous environmental factors such as age and lifestyle choices which could influence these, and each of these could in turn affect the homeostasis of retromer proteins and the proteins which interact with it.

## Conclusions

The role of the retromer in maintaining homeostasis of proteins linked with age-related diseases is clear, but the means by which age influences this function is not known. Though some work has demonstrated interactions between retromer proteins and known aging-related pathways such as insulin and mTOR, there remain very few findings of how age is related to the maintenance of this mechanism, and there is potentially conflicting evidence which suggests that retromer function may promote age-related pathways. The balance between proper sorting of proteins for reuse or degradation is intricate, but its importance is clear. Understanding how age affects the key players in this process is an essential area of study to understand neuronal homeostasis.
